# Adaptive co‐evolution of mitochondria and the Y‐chromosome: A resolution to conflict between evolutionary opponents

**DOI:** 10.1002/ece3.8366

**Published:** 2021-11-25

**Authors:** Michael J. Wade, Laurel Fogarty

**Affiliations:** ^1^ Indiana University Bloomington Indiana USA; ^2^ Max Planck Institute for Evolutionary Anthropology Leipzig Germany

**Keywords:** epistasis, inbreeding, male fertility, maternal and paternal inheritance, mitochondria, Y chromosome

## Abstract

In most species with motile sperm, male fertility depends upon genes located on the Y‐chromosome and in the mitochondrial genome. Coordinated adaptive evolution for the function of male fertility between genes on the Y and the mitochondrion is hampered by their uniparental inheritance in opposing sexes: The Y‐chromosome is inherited uniparentally, father to son, and the mitochondrion is inherited maternally, mother to offspring. Preserving male fertility is problematic, because maternal inheritance permits mitochondrial mutations advantageous to females, but deleterious to male fertility, to accumulate in a population. Although uniparental inheritance with sex‐restricted adaptation also affects genes on the Y‐chromosome, females lack a Y‐chromosome and escape the potential maladaptive consequences of male‐limited selection. Evolutionary models have shown that mitochondrial mutations deleterious to male fertility can be countered by compensatory evolution of Y‐linked mutations that restore it. However, direct adaptive coevolution of Y‐ and mitochondrial gene combinations has not yet been mathematically characterized. We use population genetic models to show that adaptive coevolution of Y and mitochondrial genes are possible when Y‐mt gene combinations have positive effects on male fertility and populations are inbred.

## Graphical abstract

Enigmatically, mitochondrial genes and Y‐linked genes share common adaptive functions essential for sperm motility and male fertility. Genes on both the Y‐chromosome and mitochondrial genes are necessary for sperm development and differentiation; deletions or mutations in either group of genes can have serious deleterious effects on adult male fertility. Nevertheless, genetic conflict is expected to make it impossible for Y‐linked and mitochondrial DNA to adaptively coevolve owing to their antagonistic, uniparental modes of inheritance. We use simple population genetic models to show that such adaptive coevolution is indeed possible when there is an epistatic fitness effect between a Y‐linked gene and a mitochondrial gene and, crucially, the population is inbreeding.

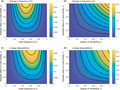

## 1 INTRODUCTION

Males appear to be an evolutionary dead end for mitochondria and other organelles with uniparental, maternal inheritance. Moreover, adaptive evolution of mitochondrial genes affecting fitness of females, the transmitting sex, can have deleterious fitness effects on males, the non‐transmitting sex (a phenomenon known as “mother's curse”; Partridge & Hurst, 1998). Although mitochondria are essential to sperm motility, mitochondria have been considered evolutionarily “…unavailable as an avenue for adaptive evolution of sperm phenotype” (Zeh, 2004, p. S307). Moreover, traits related to reproduction that are “strongly sexually dimorphic or sex limited in expression (e.g., traits related to reproduction), are predicted to be those most vulnerable to the build‐up of an underlying male‐specific mitochondrial mutation load” (Beekman et al., 2014, p. 2). As pointed out by Gemmell et al. (2004), this seemingly important role of small mtDNA mutations, having little or no effect on female fitness, in male fertility and sperm function (Ruiz‐Pesini et al., 2000) points to the existence and maintenance of sexually antagonistic cytoplasmic interactions. Further, uniparental inheritance with sex‐restricted adaptation also affects genes on the Y‐chromosome, but females escape the potential maladaptive consequences since they do not have a Y‐chromosome. Theory suggests that it is possible for Y‐linked genes that enhance male fitness to diminish female fitness indirectly via Y‐autosome gene interactions (Ågren et al., 2019a). The opposing modes of uniparental inheritance for Y‐linked and mitochondria genes could engender perpetual evolutionary genetic conflict because the maternally transmitted mitochondria are essential to male gamete function (Meiklejohn & Tao, 2010; Zeh & Zeh, 2005). In nonrandomly mating populations, the evolution of mitochondrial genes with effects on male fertility can be constrained (Unckless & Herren, 2009; Wade & Brandvain, 2009), because inbreeding creates an association between the deleterious mitochondrial effects on male fertility or viability and the fitness of the female mitochondrial lineages causing those effects. These single gene cytoplasmic models did not examine Y‐mitochondrial gene interactions or any kind of nuclear‐mitochondrial epistasis for fitness. Empirically, however, studies by Dean et al. (2015), Yee et al. (2015), and Ågren et al. (2019b) in *Drosophila melanogaster* discovered abundant mitochondrial‐Y chromosome epistasis. The findings of Ågren et al. (2019b, p. 1) are that, “In particular, genes involved in male reproduction appear to be especially sensitive to such interactions.” In this article, we examine theoretically the potential for coevolution between Y‐linked and mitochondrial genes.

The efficacy of epistatic selection acting on gene combinations depends upon the relative strengths of selection, *s*, and recombination, *r*. Positive epistatic selection creates linkage disequilibrium (LD) between genes at different loci, while subsequent recombination reduces the LD created by selection. Relative to selection on single genes, this tends to reduce the heritability of the effects of a gene combination on fitness. Positive epistatic selection acting on Y‐mt gene combinations would appear to be particularly ineffective because Y‐linked and mitochondrial genes are not only unlinked, but they are necessarily independently inherited from different sex parents. Because epistatically fertile fathers cannot pass Y‐mt gene combinations directly to their sons, it would appear to be impossible for epistatic selection to sustain Y‐mt gene combinations in affecting male fertility in LD. Theoretical studies by Connallon et al. (2018) and Ågren et al. (2019a) investigated the extent to which nuclear gene evolution could compensate for the deleterious effects of mitochondrial genes on male fitness. Such compensatory evolution is inherently epistatic. These authors investigated only one type of epistasis, compensation for a mitochondrial male fitness reduction, and did so only in randomly mating populations. Both studies investigated invasion conditions following fixation of a deleterious mitochondrial allele. When one background is fixed (here the mitochondrial haplotype), epistatic variation is converted into additive variation for the remaining segregating gene. Thus, Ågren et al. (2019a, p. 12) found that the conditions for invasion of a compensatory *y* allele are independent of mitochondrial allele frequency and that an invading *y* allele fixes whenever the compensatory effect exceeds the deleterious mitochondrial effect on males; that is, whenever the effect of the *y* on male fitness is positive, it fixes in the population. Neither study calculated LD between Y‐linked and mitochondrial alleles.

We use population genetic models to show how these potential evolutionary opponents, the mitochondrion and the Y‐chromosome, might adaptively coevolve and discuss the empirical evidence in support of our theory. We further show how our coadaptive process depends upon epistasis and inbreeding to create and sustain LD between Y‐linked and mitochondrial genes.

## 2 THE MODEL

We assume there are two Y‐linked alleles, *Y*_1_ and *Y*_2_, and two mitochondrial C alleles, *C*_1_ and *C*_2_. We let allele *Y*_1_ have a direct effect, *s*, on male fertility and an additional epistatic effect, *e*, when paired with *C*_1_. We let the eight matings (or families) in Table 2 column 10 have frequencies, *F_i_* (*i* = 1, 2, … 8) under random mating which change with inbreeding, *k*, to the values in column 11. Here, *k* determines the amount of “within family mating” or the excess frequency with which individuals with the same mitochondrial types mate. We define the frequency of *Y*_1_ *C*_1_ males as *G*_1_; that of *Y*_1_ *C*_2_ males as *G*_2_; *Y*_2_ *C*_1_ males as *G*_3_; and *Y*_2_ *C*_2_ males as *G*_4_ where genotype frequencies can be expressed as functions of the family frequencies, for example, *G*_1_ = *F*_1_ + *F*_5_. Similarly, the allele frequencies can be expressed as functions of the genotype frequencies. Here, the frequency of *Y*_1_ is *G*_1_ + *G*_2_ = *u*; that of *Y*_2_ is *G*_3_ + *G*_4_ = *v*; and the frequency of *C*_1_ is *G*_1_ + *G*_3_ = *p* and that of *C*_2_ is *G*_2_ + *G*_4_ = *q*, see Table 1 for a summary of symbols used.

**TABLE 1:** List of symbols

Symbol	Description
*s*	Direct effect of *Y*_1_ on male fertility
*e*	Epistatic effect of *Y*_1_ *C*_1_ on male fertility
*k*	Inbreeding coefficient, amount of within family mating
*G*_1_	Frequency of *Y*_1_ *C*_1_
*G*_2_	Frequency of *Y*_1_ *C*_2_
*G*_3_	Frequency of *Y*_2_ *C*_1_
*G*_4_	Frequency of *Y*_2_ *C*_2_
*p*	Frequency of *C*_1_ allele
*q*	Frequency of *C*_2_ allele
*u*	Frequency of *Y*_1_ allele
*v*	Frequency of *Y*_2_ allele

### 2.1 Random mating

We begin by considering the case of random mating (*k* = 0) and proceed to build a model that includes the effects of inbreeding and epistatis. In the random case, the mating frequencies can be found in Table 2 column 10. Here the mean fitness is given by

(1) $$W=1+us+G_{1}e.$$

**TABLE 2:** Mating table

Male	Female	Sons				Daughters		Family		
								Fitness	Frequency (random)	Frequency (with *k*)
*Y*_1_ *C*_1_	*C*_1_	1	–	–	–	1	–	1 + *s* + *e*	*F*_1_	*F*_1_ + *F*_2_ *k*
*Y*_1_ *C*_2_	*C*_1_	1	–	–	–	1	–	1 + *s*	*F*_2_	*F*_2_ (1 − *k*)
*Y*_2_ *C*_1_	*C*_1_	–	–	1	–	1	–	1	*F*_3_	*F*_3_ + *F*_4_ *k*
*Y*_2_ *C*_2_	*C*_1_	–	–	1		1	–	1	*F*_4_	*F*_4_ (1 − *k*)
*Y*_1_ *C*_1_	*C*_2_	–	1	–	–	–	1	1 + *s* + *e*	*F*_5_	*F*_5_ (1 − *k*)
*Y*_1_ *C*_2_	*C*_2_	–	1	–	–	–	1	1 + *s*	*F*_6_	*F*_6_ + *F*_5_ *k*
*Y*_2_ *C*_1_	*C*_2_	–	–	–	1	–	1	1	*F*_7_	*F*_7_ (1 − *k*)
*Y*_2_ *C*_2_	*C*_2_	–	–	–	1	–	1	1	*F*_8_	*F*_8_ + *F*_7_ *k*

We first consider the evolution of males and the change in frequency of the *Y*_1_ allele (*u*) over a single generation. Because, *u* = *G*_1_ + *G*_2_, after fertility selection (indicated by a prime ′) we have

(2) $$u^{\prime }=G_{1}^{\prime }+G_{2}^{\prime }.$$

We can expand the expressions for $G_{i}^{\prime },i\in \left\{1,2\right\}$ producing

(3) $$G_{1}^{\prime }=\frac{G_{1}p\left(1+s+e\right)+G_{2}p\left(1+s\right)}{W}=\frac{pu+pus+G_{1}pe}{W},$$

(4) $$G_{2}^{\prime }=\frac{G_{1}q\left(1+s+e\right)+G_{2}q\left(1+s\right)}{W}=\frac{qu+qus+G_{1}qe}{W}.$$

Combing these and simplifying we find that the change in *u* (Δ*u*) is given by

(5) $$\Delta u=u^{\prime }‐u=\frac{\left(u+us+G_{1}e\right)‐u\left(1+us+G_{1}e\right)}{W},$$

(6) $$\Delta u=\frac{uvs+vG_{1}e}{W}.$$

If *G*_1_ ≈ *up* (i.e., *Y*_2_ is rare and initially, LD = 0), then, we get

(7) $$\Delta u=\frac{uv\left(s+pe\right)}{W},$$

for the change in frequency of *Y*_1_. Note that the selective effect of epistasis, *e*, depends upon *p*, the frequency of the mitochondrial background with which *Y*_2_ interacts. Finally, we consider evolution in the female population by calculating the change in the frequency of *C*_1_. Following the same method as above, we get that the frequency of *C*_1_ after selection is

(8) $$p^{\prime }=\frac{p+sup+G_{1}pe}{W}.$$

And so, the change in *p* is

(9) $$\Delta p=\frac{p+\sup+G_{1}pe‐p\left(1+us+G_{1}e\right)}{W}=0.$$

This implies that in the case of random mating, Mother's Curse is still in effect despite mito‐nuclear epistasis, because evolution of the male Y does not result in evolution of the mitochondrial C. Here, fertility evolution on the Y‐chromosome could compensate for male deleterious but female beneficial mitochondrial genes, one aspect of genomic conflict typically hypothesized to characterize these uniparentally inherited genes.

### 2.2 LD in males

(10) $$\text{LD}=G_{1}‐up.$$

Assuming initial, LD = 0, that is, *G*_1_ = *up*, we find

(11) $$\Delta \text{LD}=G_{1}^{\prime }‐u^{\prime }p^{\prime },$$

(12) $$G_{1}^{\prime }=G_{1}+\Delta G_{1}\;\text{and}\;u^{\prime }=u+\Delta u\;\text{and}\;p^{\prime }=p+\Delta p.$$

Since, Δ*p* = 0 as above and Δ*u* = Δ*G*_1_ + Δ*G*_2_, we have

(13) $$\Delta \text{LD}=\Delta G_{1}‐p\Delta u=q\Delta G_{1}‐p\Delta G_{2},$$

(14) $$\Delta G_{1}=\frac{pu\left(1+s\right)‐pu\left(1+us\right)}{W}=\frac{\mathrm{uvps}}{W},$$

(15) $$\Delta G_{2}=\frac{qu\left(1+s\right)‐qu\left(1+us\right)}{W}=\frac{\mathrm{uvqs}}{W}.$$

Therefore,

(16) ΔLD=0.

Fertility selection on the Y‐allele alone, in the absence of epistasis and with random mating does not create Y‐C LD.

### 2.3 Non‐random mating

We now consider how the results above change when inbreeding structures the mating system and allows the eight possible matings (or families) to have frequencies *F_i_* with *i* = {1, …, 8} shown in Table 2, modified by *k* as shown in Table 2 column 11. We can show that the mean fitness is now given by *W* = 1 + *us* + *H*_1_ *e*, where *H_i_* = *F*_1_ + *F*_2_ *k* + *F*_5_ (1 − *k*). First, we calculate the change in *u*, the frequency of the *Y*_1_ allele. As before, we have that

(17) $$u^{\prime }=G_{1}^{\prime }+G_{2}^{\prime }.$$

Expanding the expressions for $G_{1}^{\prime }$ and $G_{2}^{\prime }$ we get the following:

(18) $$G_{1}^{\prime }=\frac{\left(F_{1}+F_{2}\right)\left(1+s\right)+e\left(F_{1}+F_{2}k\right)}{W}.$$

Similarly, for $G_{2}^{\prime }$ we get

(19) $$G_{2}^{\prime }=\frac{\left(F_{6}+F_{5}\right)\left(1+s\right)+F_{5}e\left(1‐k\right)}{W}.$$

Thus, the change in the frequency of *Y*_1_ is

(20) $$\Delta u=\frac{uvs+ve\;H_{1}}{W},$$

where *H*_1_ = *F*_1_ + *F*_2_ *k* + *F*_5_ (1 − *k*).

Again, following the same method as above, we can calculate the frequency of *C*_1_ after selection. This is given by

(21) $$p^{\prime }=\frac{\left(F_{1}+F_{2}+F_{3}+F_{4}\right)+\left(F_{1}+F_{2}\right)s+\left(F_{1}+F_{2}k\right)e}{W}.$$

And so, the overall change in the frequency of *C*_1_ after selection is

(22) $$\Delta p=\frac{s\left(q\left(F_{1}+F_{2}\right)‐p\left(F_{5}+F_{6}\right)\right)+e\left(q\left(F_{1}+F_{2}k\right)‐pF_{5}\left(1‐k\right)\right)}{W}.$$

Note that, *F*_1_ + *F*_2_ = *up*, *F*_5_ + *F*_6_ = *uq*, *F*_1_ = *G*_1_ *p*, and *F*_5_ = *G*_1_ *q*. Thus, we can simplify giving

(23) $$\Delta p=\frac{ek\left(qF_{2}+pF_{5}\right)}{W}=\frac{ek\left(up(1‐p)\right)}{W}.$$

This implies that, with inbreeding, evolution of the male Y affects evolution of female C in proportion to the epistatic effect, *e*, and the degree of inbreeding, *k*. Therefore, fertility evolution involving genes on the Y could affect mitochondrial evolution.

### 2.4 LD in males

Finally, we are interested in calculating the LD or the covariance of Y_1_ and C_1_ in males. This is defined as LD = *G*_1_ − *up*. If LD is initially 0, after selection (indicated by a prime ′) it equals LD′ or ($G_{1}^{\prime }‐u^{\prime }p^{\prime }$), where $G_{1}^{\prime }=G_{1}+\Delta G_{1}$, *u*′ = *u* + Δ*u*, and *p*′ = *p* + Δ*p*.

Noting that, if the population were initially in random mating proportions, so that *F*_4_ = *F*_7_ and *F*_2_ = *F*_5_, then $G_{4}^{\prime }=\left(F_{7}+F_{8}\right)/W=G_{4}/W$ and $G_{3}^{\prime }=\left(F_{3}+F_{4}\right)/W=G_{3}/W$ Continuing, we find that,

(24) $$W\left({\text{LD}}^{\prime }\right)=G_{1}^{\prime }G_{4}‐G_{2}^{\prime }G_{3}.$$

Using the expressions above and rearranging, we get

(25) $${\text{LD}}^{\prime }=\frac{ev\left(qG_{1}‐F_{5}\left(1‐k\right)\right)}{W^{2}}=\frac{e\;v\left(F_{5}‐F_{5}\left(1‐k\right)\right)}{W^{2}}=\frac{e\;k\left(vF_{5}\right)}{W^{2}},$$

since, *F*_5_ = *qG*_1_ (see under Equation 22).

**FIGURE 1:**
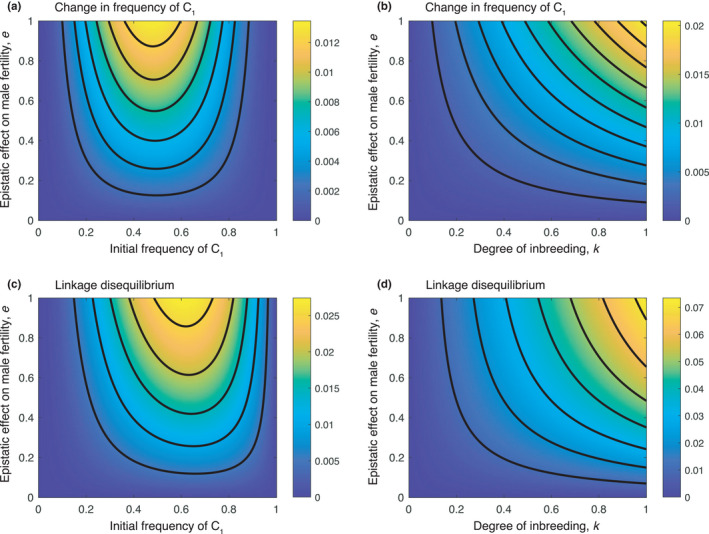
The effect of initial frequency of *C*_1_ and epistatic effect (panels a and c) and inbreeding coefficient and epistatic effect (panels b and d) on the change in *C*_1_ frequency and the LD in an inbred population. Parameters are *s* = 0.3, *k* = 0.3, *p* = 0.2, *u* = 0.2 except where varied. Note the different scales on the colorbars in each panel indicating quite different strengths of effects in each case

The LD′ reduces to 0 when either *e* or *k* = 0, as above. We illustrate the behavior of the LD as the mating system changes and the amount of inbreeding increases from 0 in Figure 1.

## 3 SUPPORTING EVIDENCE AND DISCUSSION

Many evolutionary biologists believe that mitochondrial genes and Y‐linked genes are “inexorably at odds” owing to their opposing modes of uniparental inheritance (Zeh & Zeh, 2005). Enigmatically, they share common adaptive functions essential for sperm motility and male fertility. Genes on both the Y‐chromosome and mitochondrial genes are necessary for sperm development and differentiation; deletions or mutations in either group of genes can have serious effects on adult male fertility (Dhanoa et al., 2016; Gemmell & Allendorf, 2001; Pal et al., 2017). Mitochondrial effects on male sperm performance are well known in birds (Froman & Kirby, 2005), insects (Rand et al., 2001; Yee et al., 2013), and mammals (Cardullo & Baltz, 1991), including humans (Kao et al., 1995; Moore & Reijo‐Pera, 2000; Ruiz‐Pesini et al., 2000). Because the mitochondrial genome is exclusively maternally transmitted (Frank & Hurst, 1996; Pominankowski, 1999), a selection response against male‐detrimental mitochondrial genes or for male‐beneficial genes is not possible in a randomly mating population. In human fertility studies, mitochondria are “the most important organelles for the evaluation of sperm quality*”* (Luo et al., 2013; Nakada et al., 2006) and Y chromosome microdeletions are the most common cause of human male infertility (Bansal et al., 2017).

Contrary to established evolutionary theory, our model shows that direct co‐adaptation between a Y‐linked gene and a mitochondrial gene is possible when there is Y‐mt epistasis for male fertility (*e* ≠ 0) in an inbreeding population (*k* > 0) (Figure 1). Moreover, despite their opposing modes of uniparental inheritance, selection for increased male fertility can create and sustain LD between a Y‐linked gene and a mitochondrial gene. Although evident in theory, Y‐mt LD in natural populations would likely be short lived. Because both the Y and the mitochondrion have four‐fold lower values of effective population size relative to nuclear genes, the simultaneous polymorphism, necessary for observing LD, could be rare. More often, Y‐mt epistasis might be manifest as an increase in additive variation for one component when segregating on the fixed background of the other, a phenomenon called “conversion” (Wade & Goodnight, 2006). Indeed, experimental studies of Y variation affecting male fertility against a fixed mitochondrial background, and the converse have shown such effects (see below).

There is empirical evidence to support our model assumptions. First, in *D. melanogaster*, mitochondrial and Y‐linked genes interact to affect male fertility, meaning that *e* ≠ 0. Y chromosome variation (Branco et al., 2013) disproportionately affects mitochondria‐related genes and genes whose expression is affected by mitochondrial haplotype are affected as well by Y‐chromosome variation (Guo, 2015; Rogell et al., 2014). Experimental studies in *D*. *melanogaster* have revealed Y‐mt epistatic interactions for male reproduction, including effects on male fertility and mating success (Ågren et al., 2019b; Yee et al., 2015). Although many of the data cited here are from experiments involving between‐population crosses, we note that many genetic phenomena were discovered and are studied in inter‐population crosses, including cytoplasmic inheritance, the ubiquitous arthropod microbe Wolbachia, meiotic drive and its suppressors, p‐elements and their suppressors, and Dobzhansky‐Muller incompatibilities. Notably, the latter three cases involve epistasis. For rapidly evolving phenomena within populations, polymorphisms are likely to be transient; making it is easier to discover phenomena in interspecific crosses between populations, where the same processes have arrived at different equilibria. The evidence for *adaptive epistasis* in which native Y‐mt combinations have the highest male fertility is present in some studies but not in all (Ågren et al., 2019b; Yee et al., 2013). Although, in such experiments, a finding of highest fitness on the home (or non‐novel) genetic background is typically interpreted evidence of coadaptation (see e.g., Ågren et al., 2019b for more details), some authors have hypothesized that their findings were owing to repeated cycles of mitochondrial harm to male fertility followed by autosomal restoration. This interpretation is the only one possible when the conclusions of established theory rule out direct coadaptation between a Y‐linked gene and a mitochondrial gene. Second, studies of male fertility find higher heritabilities estimated from dam than sire components, often several times higher (e.g., Beaumont et al., 1997: 0.09 sire vs. 0.31 dam). Selection experiments on sperm mid‐piece length in the mouse using within‐family selection resulted in a divergence among selected lines of 5.4 phenotypic standard deviations in 13 generations with a realized heritability of 0.76 (Woolley, 1970). Within‐family selection results in male and mitochondrial genes necessarily being selected up and down together. The design amounts to fixing the mitochondrial background and selecting on any epistatic interaction with genes still segregating. Electron microscopy showed that increases in mid‐piece length resulted in an increase in mitochondrial material in individual sperm cells, a correlated mitochondrial response, despite an absence of mitochondrial genetic variation within families. Lastly, in *Gallus domesticus*, artificial selection for increased and decreased male sperm motility, using a selection protocol that included maternal inheritance, found a strong maternal additive genetic effect (Froman et al., 2002). Molecular genetic investigation of the response to selection found that the inheritance of a single nucleotide polymorphism within the mitochondrial tRNA explained almost all of the response to selection in a sperm mobility phenotype. Although birds do not have a Y chromosome, the selection protocol represents a type of inbreeding, wherein breeding females are selected based on sperm motility performance of their brothers. Similarly, in wild populations of the Mexican house finch, *Haemorhous mexicanus*, females choose mates based on the amount of red coloration in male feathers. This coloration is produced by and concentrated by mitochondria (Ge et al., 2015). Evidence from other finch species supports this role of cytoplasmic inheritance of coloration (Evans et al., 2014). *Haemorhous mexicanus* also has high levels of inbreeding (Reinoso Pérez, 2014), which, as per our model, would permit a male trait to respond to selection based on mitochondrial variation.

## CONFLICT OF INTEREST

The authors have no conflict of interest to declare.

## AUTHOR CONTRIBUTIONS

**Michael J. Wade:** Conceptualization (lead); Formal analysis (lead); Funding acquisition (lead); Writing‐original draft (lead); Writing‐review & editing (equal). **Laurel Fogarty:** Formal analysis (supporting); Writing‐review & editing (supporting).

## Data Availability

There is no data to be archived.
